# Amelioration of High Fructose-Induced Cardiac Hypertrophy by Naringin

**DOI:** 10.1038/s41598-018-27788-1

**Published:** 2018-06-21

**Authors:** Jung Hyun Park, Hyeong Jun Ku, Jae Kyeom Kim, Jeen-Woo Park, Jin Hyup Lee

**Affiliations:** 1 Department of Food and Biotechnology, Korea University, Sejong, Korea; 2 School of Life Sciences and Biotechnology, BK21 Plus KNU Creative BioResearch Group, College of Natural Sciences, Kyungpook National University, Taegu, Korea; 3 School of Human Environmental Sciences, University of Arkansas, Fayetteville, Arkansas USA

## Abstract

Heart failure is a frequent unfavorable outcome of pathological cardiac hypertrophy. Recent increase in dietary fructose consumption mirrors the rise in prevalence of cardiovascular diseases such as cardiac hypertrophy leading to concerns raised by public health experts. Mitochondria, comprising 30% of cardiomyocyte volume, play a central role in modulating redox-dependent cellular processes such as metabolism and apoptosis. Furthermore, mitochondrial dysfunction is a key cause of pathogenesis of fructose-induced cardiac hypertrophy. Naringin, a major flavanone glycoside in citrus species, has displayed strong antioxidant potential in models of oxidative stress. In this study, we evaluated protective effects of naringin against fructose-induced cardiac hypertrophy and associated mechanisms of action, using *in vitro* and *in vivo* models. We found that naringin suppressed mitochondrial ROS production and mitochondrial dysfunction in cardiomyocytes exposed to fructose and consequently reduced cardiomyocyte hypertrophy by regulating AMPK-mTOR signaling axis. Furthermore, naringin counteracted fructose-induced cardiomyocyte apoptosis, and this function of naringin was linked to its ability to inhibit ROS-dependent ATM-mediated p53 signaling. This result was supported by observations in *in vivo* mouse model of cardiac hypertrophy. These findings indicate a novel role for naringin in protecting against fructose-induced cardiac hypertrophy and suggest unique therapeutic strategies for prevention of cardiovascular diseases.

## Introduction

Dietary fructose consumption has increased sharply in recent decades^1,2^. Recent epidemiological and biochemical studies have indicated that fructose consumption is associated with the development of insulin resistance, dyslipidemia, obesity, and diabetes^3,4^. In addition, the increase in dietary fructose intake has been paralleled by an increase in the incidence of cardiovascular diseases, including cardiac hypertrophy and hypertension^5,6^. Cardiac hypertrophy is an adaptive structural response and a crucial compensatory mechanism of the heart that occurs in response to a variety of intrinsic or extrinsic stimuli^7,8^. Consequently, prolonged cardiac hypertrophy leads to detrimental changes in cardiac function, which can lead to congestive heart failure and sudden death due to infarction^9^.

Energy supply in the form of ATP is mandatory for sustaining cardiac contractile and relaxation functions. This requirement is fulfilled by mitochondrial oxidative phosphorylation that is finely adjusted to energy needs. Emerging evidence, however, suggests that mitochondria play a very important role in the development of cardiac hypertrophy. In addition, in both human subjects and experimental models of cardiac hypertrophy, mitochondrial dysfunction is increased^10–12^. Recently, it was reported that mitochondrial dysfunction were induced by a fructose-enriched diet and mitochondrial disruption ultimately played a central role in the pathogenesis of fructose-induced cardiac hypertrophy^13^.

Mitochondria, which compose 30% of the cardiomyocyte volume^14^, are major organelles for cellular metabolism through the oxidative production of ATP and regulation of intracellular redox status^15^. Therefore, mitochondria are one of the most consistent sources of ROS during oxidative phosphorylation. This exposure of mitochondria to ROS results in oxidative damage to the organelles, which subsequently leads to impairment of mitochondrial integrity and function, thereby contributing to the development of cardiac hypertrophy, decreased cardiac function, and, ultimately, heart failure^16,17^. In particular, emerging evidence has suggested that mitochondrial ROS play a critical role in the development of fructose-induced cardiac hypertrophy^13^. Moreover, there is now mounting evidence that the pathology and progression of cardiac hypertrophy associated with heart failure involves excessive production of ROS and ROS-related oxidative stress^18^. Cardiac oxidative injury can lead to cardiomyocyte apoptosis, which contributes to the development of cardiac hypertrophy^19^.

It is well known that polyphenolic flavonoids possess antioxidant activity owing to their specific structural features that allow radical scavenging^20^. Treatment with antioxidants, such as resveratrol and those of the polyphenolic families, prevented biochemical cardiovascular changes and cardiac hypertrophy in animal models fed a high fructose diet^21,22^. Of note, naringin (4,5,7-trihydroxyflavanone-7-rhamnoglucoside), a major flavanone glycoside found in grapefruit and related citrus species, has been reported to scavenge free radicals and possesses metal-chelating and antioxidant properties^23,24^. Therefore, in the present study, we examined the activity of naringin, using *in vitro* and *in vivo* models of fructose-induced cardiac hypertrophy and dysfunction. The aim was to determine whether naringin protected cardiomyocytes against ROS-mediated apoptosis and hypertrophy by modulating fructose-induced mitochondrial dysfunction to prevent cardiac injury, representing a novel protective approach for pathological cardiac hypertrophy resulting from dietary fructose consumption.

## Materials and Methods

### Materials

The following materials were obtained from Sigma-Aldrich (St. Louis, MO, USA): fructose, naringin, 5,5′,-dithio-bis(2-mitrobenzoic acid) (DTNB), pyrogallol, propidium iodide (PI), xylenol orange, 3-(4,5-dimethylthiazol-2-yl)-2,5-di- phenyltetrazolium bromide (MTT), 3,3-diaminobenzidine (DAB), Gil no. 3 hematoxylin and eosin Y solution, and an Annexin-V-FLUOS Staining Kit. Rhodamine-123 and a JC-1 mitochondrial membrane potential probe were purchased from Thermo Fischer Scientific (Waltham, MA). Diphenyl-1-pyrenylphosphine (DPPP) and 2′,7′-dichloro-fluorescein diacetate (DCFH-DA) were purchased from Molecular Probes (Eugene, OR). CellTracker^TM^ Green CMFDA and MitoSOX Red Mitochondrial Superoxide Indicator were obtained from Invitrogen (Eugene, OR). A Seahorse XF Cell Mito Stress Test Kit was purchased from Agilent (Santa Clara, CA). The antibodies that were used in this study were acquired from Cell Signaling (Beverly, MA), Abcam (Cambridge, MA), Santa Cruz Biotechnology (Santa Cruz, CA), Virogen (Waterstown, MA), Biorbyt (Cambridge, UK), Calbiochem (San Diego, CA), and Abfrontier (Seoul, Korea).

### Cell culture

The H9c2 rat myoblastic cell line was obtained from the American Type Culture Collection (Manassas, VA). Cells were cultured with Dulbecco’s modified Eagle’s medium, supplemented with 10% fetal calf serum (FBS) and 1% penicillin/streptomycin in a humidified atmosphere of 5% CO_2_ at 37 °C. After 48 h, the medium was replaced with glucose-free DMEM with 1% FBS and 1% penicillin/streptomycin and the cells were cultured overnight. To assess the effect of fructose, cells were incubated with the indicated concentrations of fructose for the designated times.

### Animal study

The animal studies were organized according to the institutional guidelines for the care and use of laboratory animals. Six-week-old male C57BL/6 juvenile mice were housed at a consistent 22 °C temperature and 12 h light/dark cycle. All procedures were carried out in accordance with the institutional guidelines for the use and care of laboratory animals and were approved by The Institutional Animal Care and Use Committee at the Kyungpook National University. Each diet was of equal digestible energy and micronutrient-matched and was based on the American Institute of Nutrition standard rodent growth diet (AIN-76A). Rodent AIN-76A based Cereal Feed was purchased from ToDoBio (Gyeonggi-do, Korea). The control diet (Con) contained 536 g/kg starch and 100 g/kg sucrose and the 60% high fructose diet (FR) contained 36 g/kg starch and 600 g/kg fructose. Naringin (NRG) was thoroughly mixed into both the control and high fructose diets at 1.6 g/kg to give an approximate dose of 100 mg/kg/day. Mice were randomly divided into 4 groups as follows: Con, Con + NRG, FR, FR + NRG. Food intake and body weight were monitored over the 10-week treatment period until the animals were euthanized.

### Immunoblot analysis

Total protein extracts were resolved by SDS PAGE, then the proteins were transferred onto nitrocellulose membranes and probed with appropriate primary antibodies. Proteins were visualized using horseradish peroxidase-labeled anti-rabbit IgG and an enhanced chemiluminescence detection kit (Amersham Pharmacia Biotech, Buckinghamshire, UK). Protein expression (See Supplementary Information) was analyzed using Image J software.

### Assessment of cellular redox status

The concentration of intracellular hydrogen peroxide was measured by a ferric-sensitive dye xylenol orange assay. After fructose exposure, cells were harvested and suspended in xylenol orange solution (1 mM xylenol orange, 25 mM ferrous ammonium sulfate, 1 M sorbitol, 0.25 M H_2_SO_4_). Intracellular hydrogen peroxide was analyzed using a spectrophotometer at 560 nm. Intracellular ROS generation was also measured using an oxidant-sensitive fluorescent probe, DCFH-DA. After fructose exposure, cells were treated with 10 μM DCFH-DA for 30 min at 37 °C and ROS levels were analyzed using an FACS CALIBUR flow cytometer (BD Biosciences, San Jose, CA). Cellular GSH levels were measured using the GSH-sensitive fluorescent dye, CMFDA. After 24 h of fructose exposure, cells were stained with 5 µM CMFDA for 30 min at 37 °C. Fluorescent intensity was analyzed using a Zeiss Axiovert 200 inverted microscope. Thiobarbituric acid-reactive substances (TBARS) were used to measure lipid preoccupation. After the indicated exposure, cells were washed in PBS and harvested. Cell extracts were mixed with 1 mL TBA solution (0.375% thiobarbituric acid in 0.25 N HCl containing 15% (w/w) tricoloroacetic acid). Lipid peroxidation was detected using fluorescent probe DPPP dye. After fructose exposure, cells were incubated with 5 µM DPPP probe at 37 °C in the dark. The light intensity was evaluated using the Zeiss Axiovert 200 inverted microscope.

### Mitochondrial membrane potential and ROS

To evaluate mitochondrial redox status, a JC-1 fluorescent probe was used to detect mitochondrial membrane potential. After 24 h of fructose exposure, cells were washed with PBS and incubated with 5 µM of JC-1 for 20 min at 37 °C. The ratio of the green and red fluorescent intensities was used as an indicator of mitochondrial membrane potential^25^. Mitochondrial membrane permeability transition (MPT) was visualized with rhodamine-123 as previously described^26^. After 24 h of fructose exposure, cells were treated with 5 μM rhodamine-123 for 20 min at 37 °C. The fluorescent intensity was measured and analyzed. The mitochondrial ROS level was measured using MitoSOX red fluorescent dye. Cells were exposed to high fructose for 6 h, then washed with PBS and incubated with 10 μM MitoSOX Red for 20 min at 37 °C. Each fluorescent image was evaluated using the Zeiss Axiovert 200 inverted microscope.

### Oxygen consumption rate (OCR)

The mitochondrial OCR was evaluated using an Agilent Seahorse XF Cell Mito Stress Test Kit following the manufacturer’s protocol, which included a phased addition of oligomycin, FCCP, and the mix of rotenone and antimycin A. These serial additions represented individual parameters for basal respiration, proton leak, maximal respiration, and spare respiratory capacity^27^. Each sequential parameter was measured using an XF24 analyzer (Seahorse Bioscience, North Billerica, MA).

### Histological analysis

H9c2 cells were fixed with 3% formaldehyde in PBS for 15 min at room temperature, gently washed twice with PBS. The methanol permeabilization step was performed with 100% ice-cold methanol at −20 °C for 10 min. After fixation, the immunostaining step was performed as previously described^28^.

### Statistical analysis

Statistical analyses were performed by two-tailed *t* tests. Results are shown as means ± S.D.

## Results and Discussion

### Effect of naringin on fructose-induced cardiomyocyte hypertrophy

**Figure 1:**
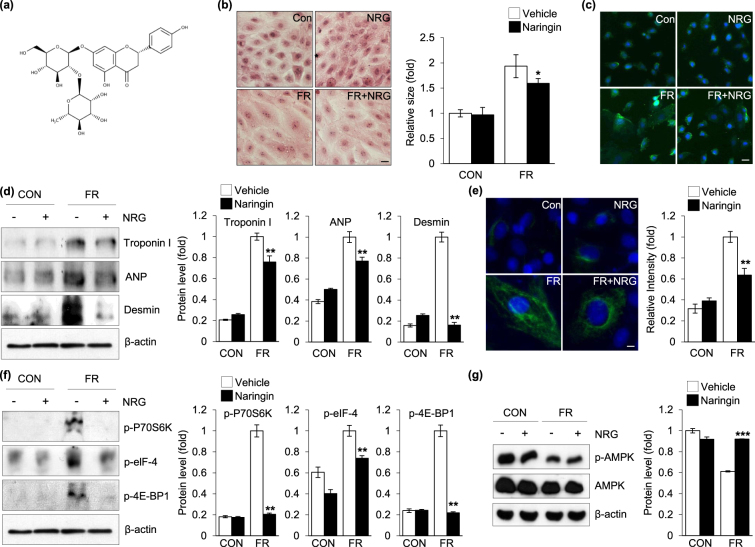
Protective effect of naringin against fructose-induced cardiomyocyte hypertrophy. (**a**) The structure of naringin. (**b**) Representative hematoxylin and eosin stained image of H9c2 cells. Cells were exposed to fructose (50 mM, 24 h) in the presence and absence of naringin (80 µM, 2 h). The histogram represents H9c2 cell size which was quantified after H&E staining. (**c**) Representative images of immunofluorescece staining of α-actinin in H9c2 cells. Nuclei were counterstained with Hoechst33342. (**d**) Immunoblot analysis of intracellular expression of hypertrophic markers in H9c2 cells. Actin was used as a loading control. Quantifications of the levels of troponin I, ANP, and desmin normalized to actin are shown. (**e**) Immunofluorescence analysis of vimentin expression in H9c2 cells. Histograms represent the quantification of fluorescence intensity. (**f**) Immunoblot analysis of muscle protein synthesis marker levels in the H9c2 cells. Actin served as a loading control. The proteins levels were normalized to the actin level. (**g**) Immunoblot analysis for the status of AMPK activation. H9c2 cells were treated with fructose (50 mM, 24 h) in the presence and absence of naringin (80 µM, 2 h). Actin was used as a loading control. Quantification of p-AMPK levels as normalized to AMPK is shown. Data are presented as the mean ± S.D. of three separate experiments (*P < 0.05, **P < 0.01, ***P < 0.001 vs. fructose treated group). Scale bar in images indicates 20 μm. Con: control group, FR: fructose group, NRG: naringin treatment group compared with vehicle group.

To determine whether naringin (Fig. 1a) directly inhibited the enlargement of cardiomyocytes, a hallmark of cardiac hypertrophy, by fructose, we treated these cells with 50 mM of fructose in the presence or absence of naringin for 24 h. The results indicated that the cardiomyocyte cell size was markedly increased after fructose treatment, whereas the addition of naringin led to a decrease in the fructose-induced enlargement of the cardiomyocytes (Fig. 1b). To further verify the inhibitory effects of naringin on the fructose-induced enlargement of cardiomyocytes, α-actinin, sarcomeric α-cardiac-actinin, was used to analyze the morphology and enlargement of cardiomyocytes. As shown in Fig. 1c, naringin inhibited the fructose-induced enlargement of cells. To further assess the effects of naringin on fructose-induced cardiomyocyte hypertrophy, we measured the expression of various markers of cardiomyocyte hypertrophy, myocardial cytoskeleton proteins such as troponin I and desmin. A substantial increase in the abundance of troponin I and desmin was observed compared with those in the control cells when cardiomyocytes were exposed to fructose (Fig. 1d). However, naringin significantly inhibited the expression of key proteins involved in cardiomyocyte hypertrophy compared to that in control cells when the cells were treated with fructose. We also evaluated the effect of naringin on ANP expression, another marker of cardiomyocyte hypertrophy^29^. The data were further corroborated by the immunofluorescence analysis of vimentin expression by fluorescence microscopy (Fig. 1e). These results suggested that treatment with naringin resulted in reduced development of hypertrophy in the cardiomyocytes after exposure to fructose. It is well established that mTOR protein kinase plays a critical role in regulating the expression of the proteins involved in cardiomyocyte hypertrophy stimulated by nutrients such as fructose^30–33^. Therefore, to examine the contribution of naringin to mTOR regulation of myocardial cytoskeletal protein expression, we treated cardiomyocytes with fructose in the presence or absence of naringin and analyzed the activity of downstream signaling through the mTOR pathway^30–32^. As expected, mTOR-mediated phosphorylation and activation of the proteins were enhanced compared to those in the control cells and the enhanced mTOR activity seen in the cells was drastically reduced with naringin treatment (Fig. 1f). This suggested that naringin suppressed the fructose-induced increase in myocardial cytoskeletal protein expression by intervening in the mTOR signaling pathway. We next attempted to elucidate the mechanism by which naringin treatment led to a down-regulation in myocardial cytoskeleton protein expression through the mTOR signaling pathway after exposure of the cardiomyocytes to fructose. We evaluated AMPK as a potential candidate to establish a link between mTOR activity and naringin because mTOR kinase is a direct downstream target of AMPK^34,35^. In addition, it was recently reported that the phosphorylated active form of AMPK was regulated after exposure to fructose^36,37^. Figure 1g indicates that AMPK inactivation in cells exposed to fructose compared to that in control cells and significant elevation of phosphorylated AMPK levels when the cells were treated with naringin.

### Modulation of mitochondrial function by naringin in fructose-exposed cardiomyocytes

**Figure 2:**
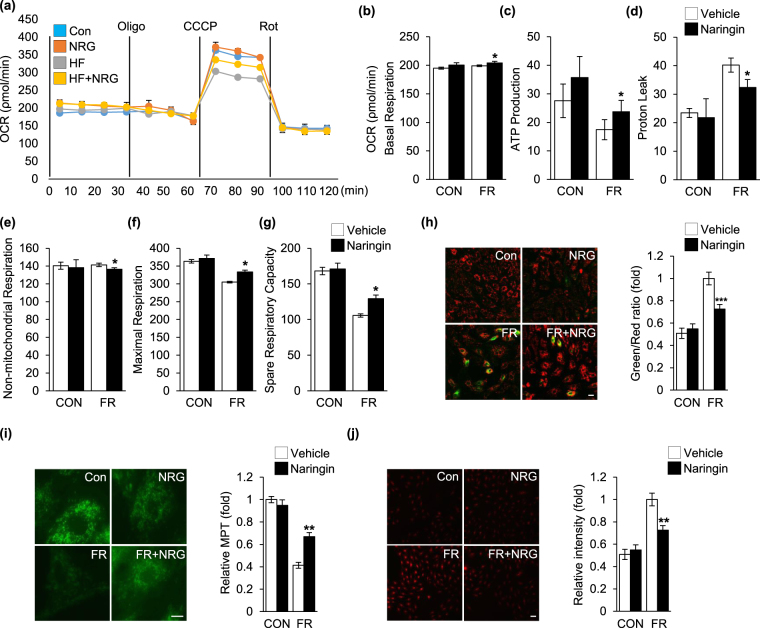
Regulation of mitochondrial function by naringin in fructose-exposed cardiomyocytes. (**a**) The mitochondrial oxygen consumption rate (OCR) was analyzed to assess the mitochondrial profile. Graphs show the quantification of the mitochondrial basal respiration rate (**b**), ATP production (**c**), proton leak (**d**), non-mitochondrial respiration (**e**), maximal respiration rate (**f**), and spare respiratory capacity (**g**). (**h**) Mitochondrial membrane potential (MP) in H9c2 cells was evaluated by JC-1 fluorescence level. The histogram represents the quantification of MP as a ratio of JC-1 (green/red) in the different treatment groups. (**i**) The rhodamine-123 fluorescence level for evaluation of mitochondrial membrane permeability transition (MPT). Histograms represent the quantification of fluorescence intensity. (**j**) The MitoSOX fluorescence level for evaluation of mitochondrial ROS generation in H9c2 cells. The histogram represents quantification of fluorescence intensity. Results are shown as the mean ± S.D. of three separate experiments (*P < 0.05, **P < 0.01, ***P < 0.001 vs. fructose treated group). Scale bar in images indicates 20 μm. Con: control group, FR: fructose group, NRG: naringin treatment group compared with vehicle group.

Recently, it was reported that by sensing the energy homeostasis from mitochondria, AMPK played a key role in intracellular energy metabolism and mitochondrial dysfunction that preceded the depression of AMPK activity^38^. To examine the effect of naringin on impaired mitochondrial function after exposure of the cardiomyocytes to fructose, we studied mitochondrial OCR in the cells (Fig. 2a)^39,40^. Figure 2b indicates that fructose exposure caused a decrease in basal respiration compared with that in control cells. In addition, fructose exposure resulted in a decrease in ATP-linked oxygen consumption (Fig. 2c). Fructose treatment markedly increased proton leak compared with that in control cells (Fig. 2d), but did not have any significant effect on non-mitochondrial respiration (Fig. 2e). With regard to maximal and spare mitochondrial respiratory capacity, we found that fructose exposure caused a significant decrease in the respiratory capacity of the cells. However, naringin clearly ameliorated fructose-induced mitochondrial disruption in cardiomyocytes (Fig. 2f and g). Fructose exposure resulted in the impairment of mitochondrial membrane potential compared to that in controls, which is also an important parameter used to assess the functional state of these organelles^41,42^. However, the impaired mitochondrial membrane potential seen in the cells was significantly reduced compared to that seen in the control cells when the cells were treated with naringin (Fig. 2h and i). Accumulating evidence suggests that mitochondrial ROS play a central role in the alteration and impairment of mitochondrial integrity and function^43^. In particular, a recent study reported that fructose intervention resulted in an increase in mitochondrial ROS levels, which was closely associated to the development of fructose-induced hypertrophy^13^. Consequently, we examined the effect of naringin on the generation of mitochondrial ROS after exposure of the cardiomyocytes to fructose. As shown in Fig. 2j, significantly more mitochondrial ROS were observed in cells exposed to fructose, but naringin significantly suppressed the increase in the production of mitochondrial ROS in fructose-treated cells. Taken together, the data indicate that naringin inhibited the increase in mitochondrial ROS levels after cellular exposure to fructose and thereby protected against fructose-induced cardiomyocyte hypertrophy through regulation of the AMPK-mTOR signaling axis.

### Effect of naringin on fructose-induced cardiomyocyte apoptosis

**Figure 3:**
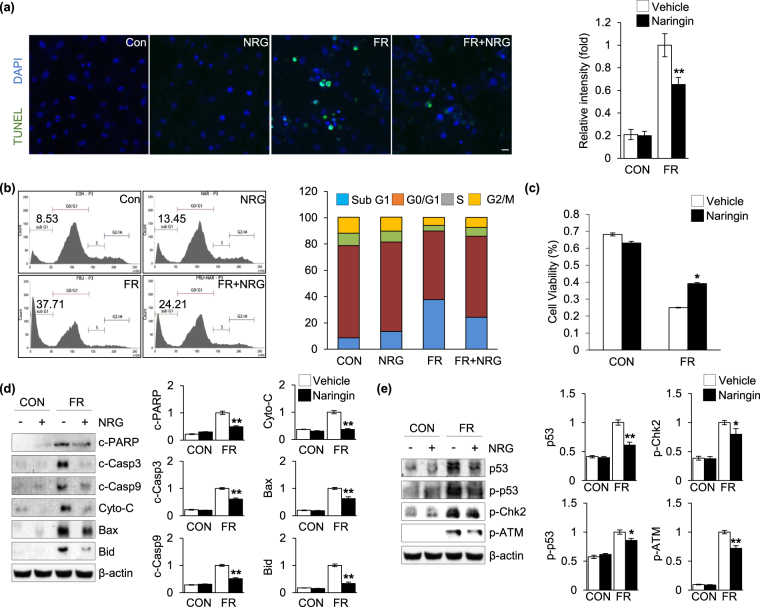
Naringin inhibited fructose-induced cardiomyocyte apoptosis. (**a**) TUNEL staining of H9c2 cells after fructose exposure in the presence and absence of naringin. Nuclei were counterstained with Hoechst33342. Histograms represent the quantification of fluorescence intensity. (**b**) Analysis of the cell cycle by flow cytometry. The bar graph shows the percentages of cells in the Sub G1, G0/G1, S, and G2/M phases. (**c**) Cell viability of H9c2 cells (MTT assay). The graph shows the comparison of cell viability between naringin treated cells and untreated cells after fructose exposure. (**d**) Immunoblot analysis of proteins from H9c2 cells related to cell apoptosis. Actin was used as a loading control. Quantification of the levels of the indicated proteins is normalized to actin expression. (**e**) Immunoblot analysis of proteins related to the ATM-mediated signal activation of H9c2 cells. Actin was used as a loading control. The proteins levels were normalized to the actin level. Data are shown as the mean ± S.D. of three separate experiments (*P < 0.05, **P < 0.01 vs. fructose treated group). Scale bar in images indicates 20 μm. Con: control group, FR: fructose group, NRG: naringin treatment group compared with vehicle group.

Cardiomyocyte apoptosis has also been shown to play a critical role in the development of fructose-induced cardiac hypertrophy^44^. We detected DNA fragments using a terminal deoxynucleotidyl transferase dUTP nick end labeling (TUNEL) assay to visualize apoptotic cell death in the cardiomyocytes. Figure 3a indicates that fructose elicited a remarkably enhanced apoptotic response in the cardiomyocytes and, importantly, this increased sensitivity to apoptosis was markedly reduced by naringin treatment. In addition, we examined the effect of naringin and fructose on the perturbation of the cell cycle because the induction of apoptosis is presumably mediated by the regulation of the cell cycle. Like the results in Fig. 3a, when the cells were exposed to fructose, the subG_1_ population, which indicates the number of apoptotic cells, was markedly increased compared with that in control cells. In addition, naringin treatment led to a substantial decrease in fructose-induced apoptotic cell damage (Fig. 3b). Importantly, the decreased sensitivity to apoptosis after naringin treatment was reflected in the cell viability results (Fig. 3c). We next examined the effect of naringin on the modulation of apoptotic marker proteins in the cardiomyocytes after exposure to fructose. Activation of caspase-3 and caspase-9was more pronounced when the cells were exposed to fructose. The formation of fragments indicative of proteolytic PARP cleavage, a pro-apoptotic marker, significantly increased in cells exposed to fructose compared to that in control cells. The levels of pro-apoptotic proteins, such as Bax, Bid, and cytochrome c, were also significantly increased after exposure to fructose. However, the increased apoptotic activities seen in the cells were drastically reduced compared to that seen in control cells after naringin treatment (Fig. 3d). To further evaluate the effect of naringin on the pro-apoptotic signaling pathway in fructose-exposed cardiomyocytes, we examined the phosphorylation and activation of the ATM-Chk2-p53 signaling axis, which plays a crucial role in regulating apoptosis in response to various cellular stressors, such as genotoxic and oxidative stress^45–47^. Figure 3e indicates that fructose significantly increased the phosphorylation of ATM, Chk2, and p53 transcription factor in the cells compared to that in controls after exposure to fructose, but naringin effectively inactivated the phosphorylation of the components involved in this signaling pathway. This suggested that inactivation of the ATM-Chk2-p53 signaling axis contributed, at least in part, to the preventive effect of naringin against fructose-induced cardiomyocyte apoptosis.

### Modulation of intracellular ROS production by naringin in fructose-exposed cardiomyocytes

**Figure 4:**
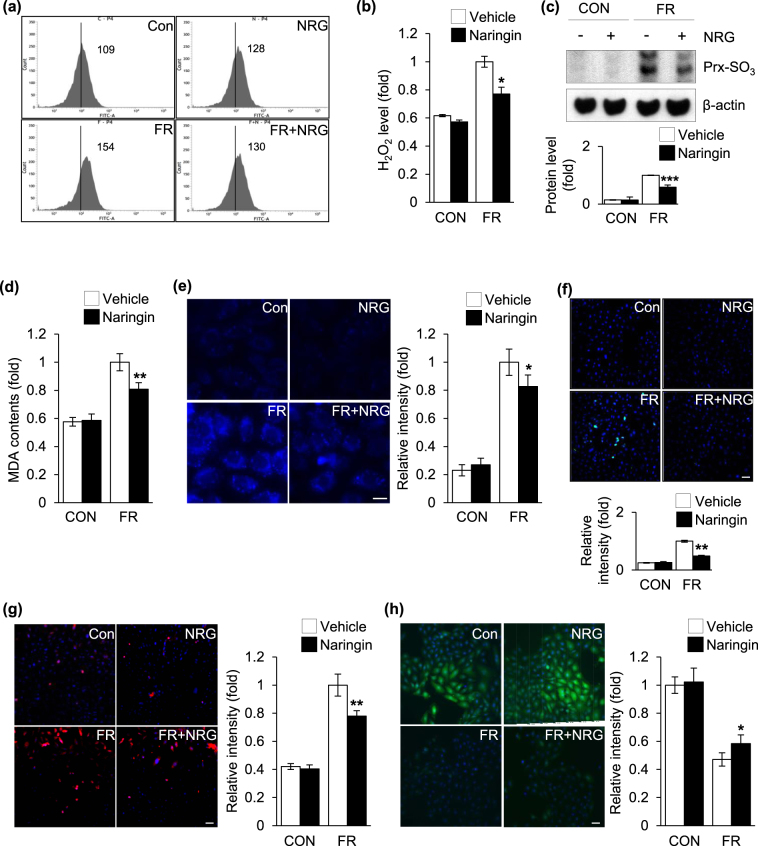
Naringin suppressed intracellular ROS production in fructose-exposed cardiomyocytes. (**a**) Flow cytometric analysis of DCFH-DA fluorescence levels for intracellular hydrogen peroxide production in H9c2 cells after fructose exposure. (**b**) Intracellular hydrogen peroxide production was measured using a xylenol orange assay. (**c**) Immunoblot analysis of Prx-SO_3_ levels in H9c2 cells. Actin was used as a loading control. Quantification of the protein levels normalized to actin is shown. (**d**) TBARS assay for assessment of the level of accumulated MDA in H9c2 cells. (e) DPPP fluorescence level for assessment of lipid peroxidation levels in H9c2 cells. Histograms represent the quantification of fluorescence intensity. (**f**) Representative images of the 8-OHdG (Green) immunohistochemical analysis measuring oxidative DNA damage. Double staining for nuclear morphology was performed with Hoechst 33342. Histograms represent the quantification of fluorescence intensity. (**g**) Immunohistochemical staining for the levels of S-glutathionylated adducts. Histograms represent the quantification of fluorescence intensity. (H) CMFDA fluorescence level for evaluating intracellular GSH levels in H9c2 cells. Histograms represent the quantification of fluorescence intensity. All data are presented as the mean ± S.D. of three separate experiments (*P < 0.05, **P < 0.01, ***P < 0.001 vs. fructose treated group). Scale bar in images indicates 20 μm. Con: control group, FR: fructose group, NRG: naringin treatment group compared with vehicle group.

It has been well established that coordination between cytoplasmic and nuclear activities is vital for the cellular stress response, including the apoptotic response, and several signaling molecules and transcription factors, such as ATM kinase and the tumor suppressor p53, are regulated by intracellular ROS during this intracellular communication^48,49^. Of interest, emerging evidence suggests that exposure to fructose contributes to an increase in intracellular ROS production in cardiomyocytes, which plays a pivotal role in the pathogenesis of fructose-induced cardiac hypertrophy^14,50^. Antioxidant treatment with agents such as tempol and resveratrol prevented biochemical cardiovascular changes and cardiac hypertrophy in fructose-fed animal models^22,51^. Naringin, a major grapefruit flavonoid, possessed strong antioxidant and anti-inflammatory activities both *in vitro* and *in vivo*^23,24^. This strongly suggested that the antioxidant activity of naringin modulated cellular redox status in the cardiomyocytes after exposure to fructose. This compound is therefore responsible for the inhibitory regulation of this signaling pathway, which contributes to its beneficial role in the down-regulation of cardiomyocyte apoptosis. Therefore, we examined the effect of naringin on intracellular ROS production after exposure of the cardiomyocytes to fructose. Increased ROS levels were observed when the cells were treated with fructose, but naringin significantly suppressed the increased ROS production in fructose-treated cells (Fig. 4a and b). In addition, the peroxiredoxin (Prx)-SO_3_ level, a marker for oxidative damage to the antioxidant enzyme Prx^52^, was also increased when the cells were exposed to fructose, whereas naringin treatment efficiently suppressed the increased ROS formation (Fig. 4c). The lipid peroxidation level, an indicative marker of cellular oxidative damage^53,54^, was increased in cells exposed to fructose. This increase was markedly reduced by naringin treatment (Fig. 4d). Similar results were obtained with DPPP, which is a fluorescent probe suitable for monitoring lipid peroxidation within the cell membrane (Fig. 4e)^55^. In addition, the fluorescent intensity reflective of endogenous 8-OH-dG levels, DNA lesion generated by intracellular ROS^53^, was significantly increased in fructose-treated cells compared with control cells and naringin treatment led to a marked reduction in the occurrence of the oxidative DNA damage induced by fructose (Fig. 4f). S-glutathionylation is a posttranslational modification of protein sulfhydryl groups that occurs under the intracellular ROS formation^53^. Immunocytochemistry, using an anti-S-glutathionylation antibody, revealed higher levels of the glutathionylated proteins when the cells were exposed to fructose. This increase was significantly diminished after naringin treatment (Fig. 4g). Opposing results were obtained with the GSH-sensitive fluorescent dye, CMFDA, determination of a change in which provides an alternative method for monitoring intracellular ROS production (Fig. 4h)^54^. The data revealed that naringin treatment led to a substantial reduction in the increase in intracellular ROS generation in cardiomyocytes exposed to fructose. Taken together, the data in Fig. 4 indicate that treatment with naringin, resulting in the suppression of elevated cellular ROS production, regulated the ROS-dependent ATM-mediated p53 axis in cardiomyocytes after exposure to fructose.

### Effect of naringin on fructose-induced cardiac hypertrophy

**Figure 5:**
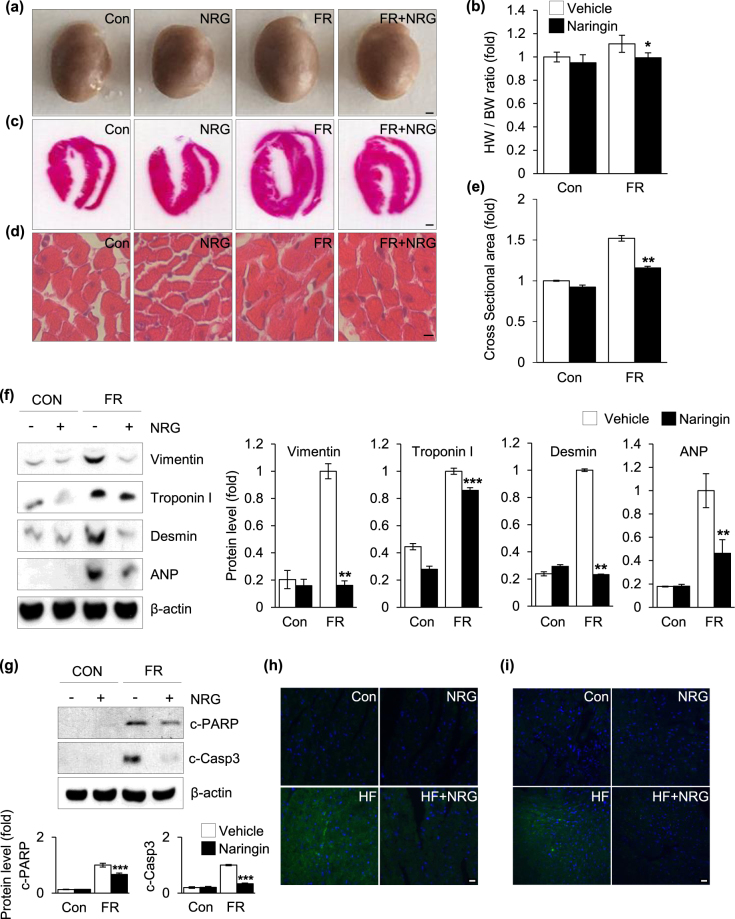
The protective effect of naringin on fructose-induced cardiac hypertrophy *in vivo*. (**a**) Macroscopic appearance of cardiac hypertrophy from mice groups. (**b**) Heart weight/body weight (HW/BW) ratios. (**c**) Representative hematoxylin and eosin stained images of heart sections. (**d**) Section images of myocardial tissue with H&E staining. (**e**) Quantification of cross-sectional area of hearts stained with H&E. (**f**) Immunoblot analysis of hypertrophy markers. Histograms represent quantification of the levels of protein normalized to actin. (**g**) Immunoblot analysis of cleavage of PARP and caspase-3. Actin was used as a loading control. Quantification of the protein levels normalized to actin is shown. (**h**,**i**) Representative immunohistochemical images of cleaved-PARP and cleaved-caspase-3 in heart sections. Nuclei were counterstained with Hoechst 33342. Each value is presented as the mean ± S.D. from three to four independent experiments (*P < 0.05, **P < 0.01, ***P < 0.001 vs. fructose treated group). Scale bar in images indicates 20 μm. Con: control group, FR: fructose group, NRG: naringin treatment group compared with vehicle group.

As a further proof of principle that naringin can be used as a therapeutic modality, the potential role of naringin in fructose-induced cardiac hypertrophy was investigated using a high-fructose diet mouse model of cardiac hypertrophy. The mouse model was established in 8-week-old male C57BL/6J mice fed a high-fructose diet with or without naringin (100 mg/kg per day) for 10 weeks. After the administration period, the efficacy of naringin in the treatment of fructose-induced cardiac hypertrophy was examined by performing *in vivo* evaluations of cardiac structure and a histological investigation of the paraffin sections from the mouse hearts. There was an approximately 40% enlargement in the hearts harvested from the mice fed the high-fructose diet compared to that in control mice fed a normal chow diet. The enlargement in the heart was mitigated by naringin treatment (Fig. 5a). In addition, the heart-to-body weight ratio (HW/BW) was higher in mice that consumed the high-fructose diet than in the control group, but naringin treatment attenuated the increased HW/BW ratio (Fig. 5b), indicating that naringin treatment inhibited high-fructose-induced cardiac hypertrophy. Morphological analysis of the transverse sectioned, H&E-stained hearts also revealed that a high-fructose diet caused adverse structural remodeling, but the morphological changes in the cardiac structure and cardiac myocytes were reduced after treatment with naringin to a level comparable to that seen in control mice (Fig. 5c and d). In addition, the cardiac myocyte cross-sectional area was significantly larger in the high-fructose-fed mice than in the control mice (Fig. 5e). However, administration of naringin preferentially ameliorated the pathological hypertrophic phenotypes of cardiomyocytes induced by high-fructose intervention (Fig. 5d and e). To further assess the association between naringin and fructose-induced cardiac hypertrophy, we measured the expression of various markers of cardiomyocyte hypertrophy. Immunoblot analysis detected substantial increases in the expression of vimentin, troponin I, desmin, and ANP in the heart tissue of the high-fructose-fed mice compared to those in the heart tissue of the control mice. This increase was significantly reduced after naringin treatment (Fig. 5f). *In vivo* measurement of apoptotic responses in the cardiomyocytes after naringin treatment indicated that there was a large decrease in the strong positive apoptosis signals within the cardiomyocytes of the mice after high-fructose administration, as measured by the extent of proteolytic cleavage of PARP (Fig. 5g and h) and caspase 3 (Fig. 5g and i). Therefore, the results obtained from the *in vivo* mouse model of cardiac hypertrophy supported those from the *in vitro* experiments that indicated that naringin inhibited fructose-induced hypertrophy and cardiomyocyte apoptosis.

In conclusion, the present study demonstrated that naringin protected against fructose-induced cardiac hypertrophy through the regulation of multiple mechanisms as follows: (1) inhibition of cardiomyocyte hypertrophy by regulating the AMPK-mTOR signaling axis, which resulted from the modulation of mitochondrial ROS generation and function; and (2) suppression of apoptotic cardiomyocyte death via regulation of ROS-dependent ATM-mediated p53 signaling. Our findings, therefore, provide the first evidence that naringin directly antagonizes fructose-induced apoptosis and cardiomyocyte hypertrophy and is a central regulator of cellular redox balance and mitochondrial redox status and function. Therefore, our results suggest the potential development of naringin as a therapeutic agent in the treatment or prevention of cardiovascular diseases such as cardiac hypertrophy.

## Electronic supplementary material

Supplementary Info
