# Supplementary material for: Amelioration of High Fructose-Induced Cardiac Hypertrophy by Naringin
Source: Sci Rep. 2018 Jun 21;8:9464. doi: 10.1038/s41598-018-27788-1 (PMC6013481; doi:10.1038/s41598-018-27788-1)
Supplement: Supplementary file 1 — Supplementary Info [file 41598_2018_27788_MOESM1_ESM.pdf]

Amelioration of High Fructose-Induced Cardiac Hypertrophy by Naringin

Jung Hyun Park[a], Hyeong Jun Ku[b], Jae Kyeom Kim[c], Jeen-Woo Park[b,*], Jin Hyup Lee[a,*]

[a]Department of Food and Biotechnology, Korea University, Sejong, Korea, [b]School of Life Sciences and Biotechnology, BK21 Plus KNU Creative BioResearch Group, College of Natural Sciences, Kyungpook National University, Taegu, Korea, [c]School of Human Environmental Sciences, University of Arkansas, Fayetteville, Arkansas, USA

Running Title: Naringin protects fructose-induced cardiac hypertrophy

*Correspondence should be addressed to Jin Hyup Lee; jinhyuplee@korea.ac.kr and Jeen-Woo Park; parkjw@knu.ac.kr

Supplementary Figures

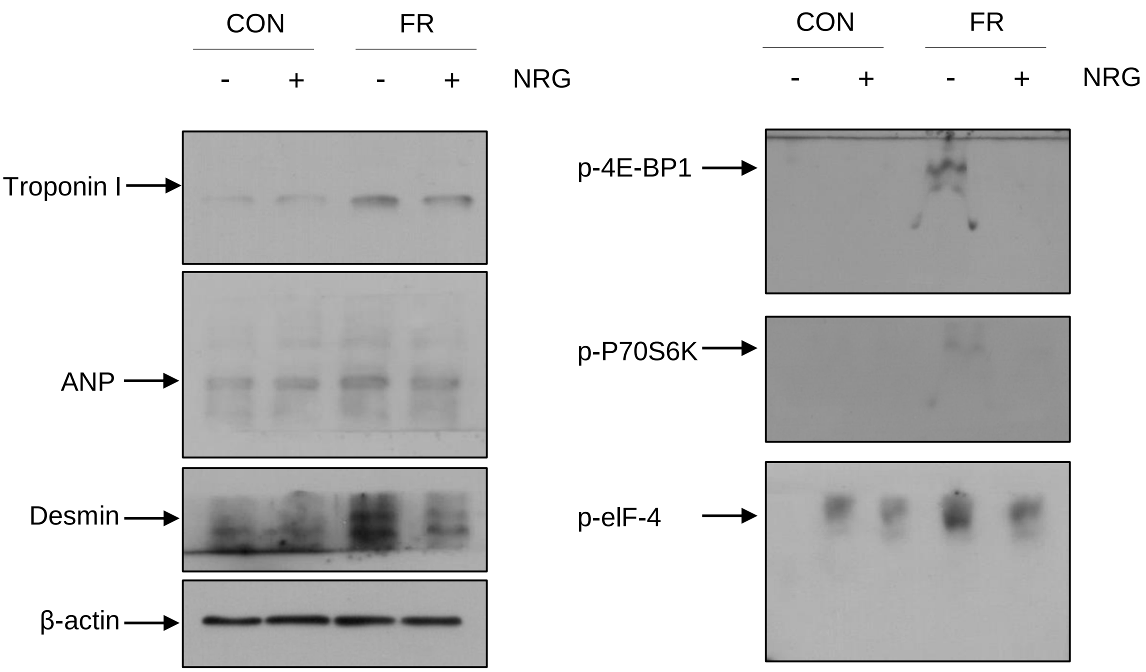

Supplementary Figure S1. The original bands for Figure 1d and f in the main manuscript.

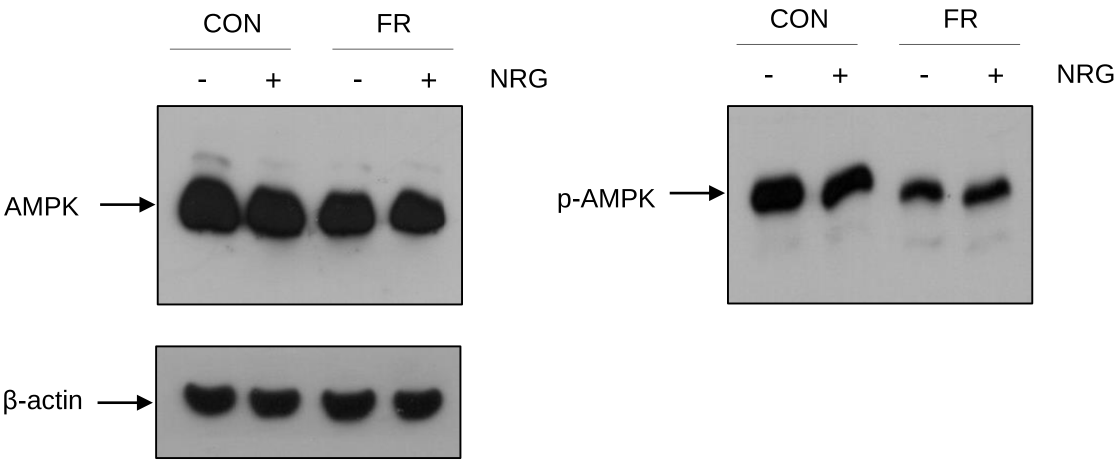

Supplementary Figure S2. The original bands for Figure 2a in the main manuscript.

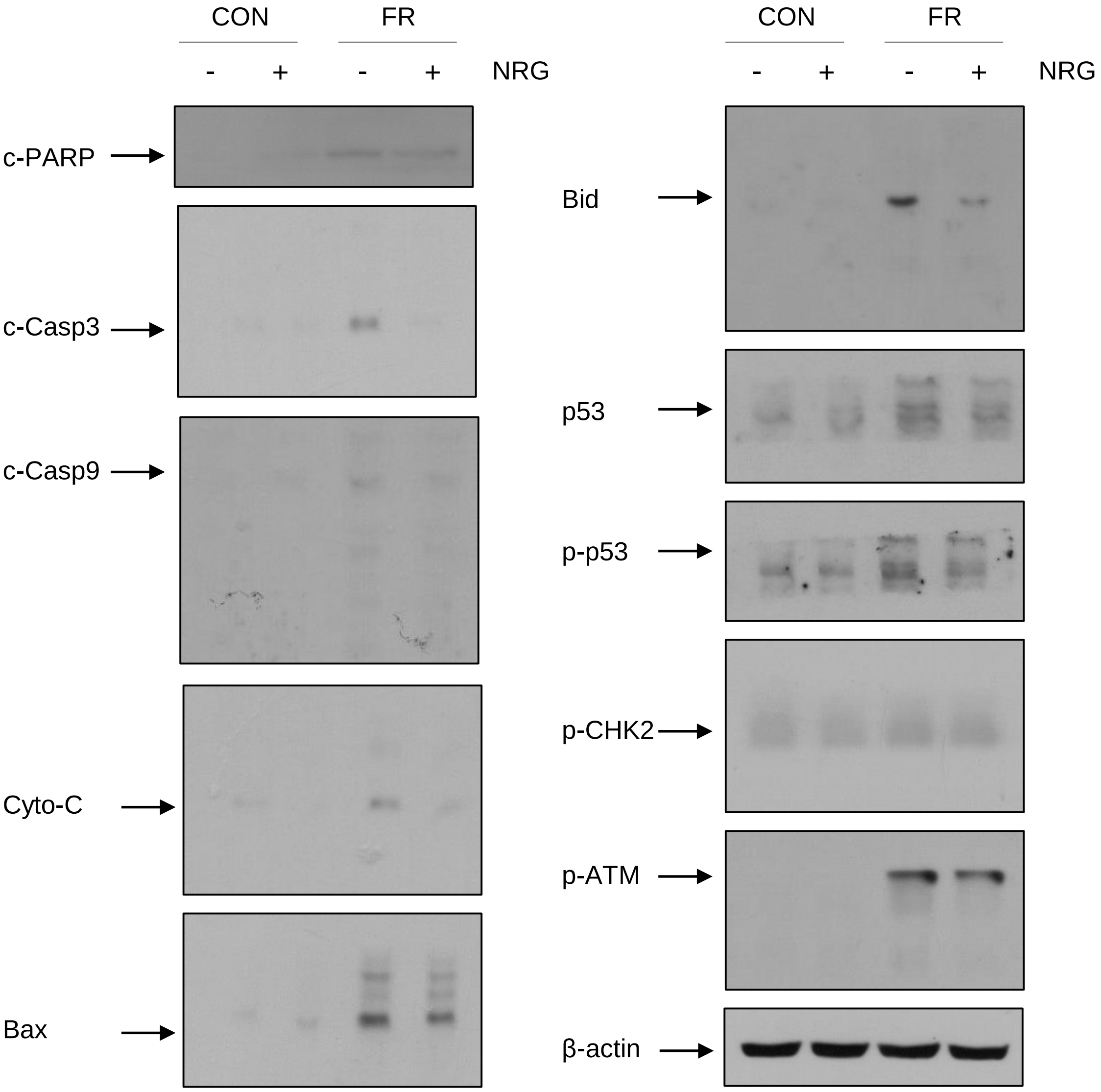

Supplementary Figure S3. The original bands for Figure 3e and f in the main manuscript.

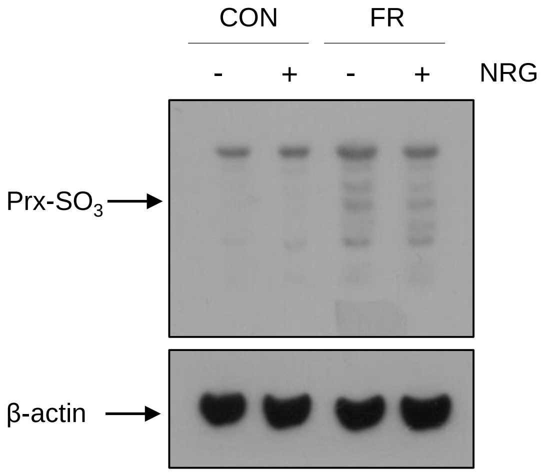

Supplementary Figure S4. The original bands for Figure 4c in the main manuscript.

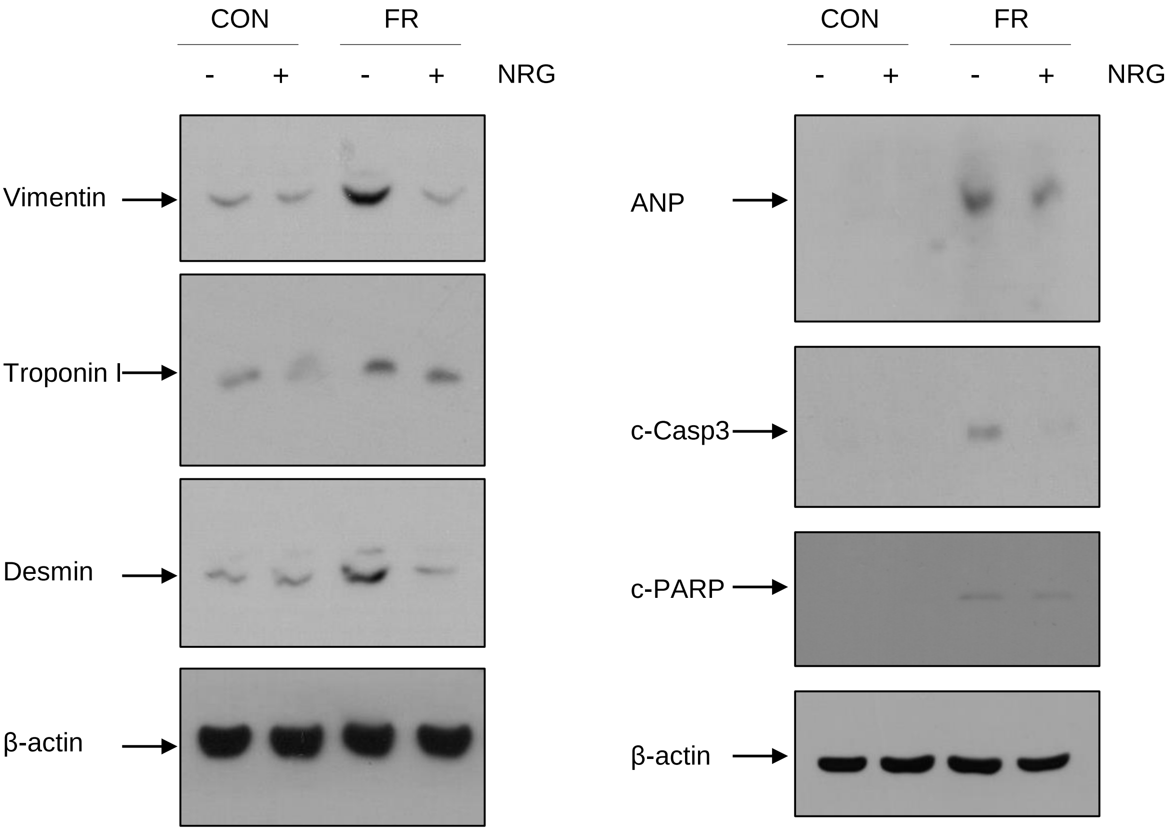

Supplementary Figure S5. The original bands for Figure 5f and g in the main manuscript.
